# Lipid‐Based Modulation of Neurogenesis: DHA‐H and HPA Promote Proliferation and Differentiation in Alzheimer's Models

**DOI:** 10.1002/alz70859_106958

**Published:** 2025-12-26

**Authors:** Enrico Castroflorio, Joan Cabot, Margalida Suau, Paula Fernández‐García, Victoria Lladó, Pablo V. Escriba, Manuel Torres

**Affiliations:** ^1^ Laminar Pharmaceuticals S.A., Palma Spain; ^2^ University of the Balearic Islands, Palma Spain

## Abstract

**Background:**

Lipids are fundamental to neuronal function, and disruptions in lipid homeostasis are increasingly recognized as key contributors to neurodegenerative diseases such as Alzheimer's disesase. While lipid dysregulation is known to alter protein function, the therapeutic potential of lipid‐based interventions remains largely unexplored.

**Methods:**

Neurospheres culture, immunocytochemistry, immunohistochemistry, inmunofluorescence labeling, confocal microscopy, western‐blot, qPCR.

**Results:**

Here, using 5xFAD mice and primary neurospheres, we investigate the effects of a hydroxylated derivative of docosahexaenoic acid (DHA‐H) and its metabolite heneicosapentaenoic acid (HPA) on neuronal proliferation and differentiation. We show that DHA‐H and HPA enhance proliferation, as evidenced by increased expression of Ki‐67, Sox2, and Nestin, alongside BrdU incorporation in vitro. Confocal microscopy and Western blot analyses further reveal upregulation of neuronal differentiation markers, including Doublecortin, NeuroD1, and PSA‐NCAM. In vivo, treatment of WT and 5xFAD mice promotes neuronal progenitor cell proliferation in the dentate gyrus, as indicated by elevated staining of phospho‐Histone H3 levels. Moreover, we identify GPR37 as a potential receptor mediating these effects, with its expression upregulated following treatment. This is accompanied by alterations in MAPK and mTOR signaling pathways, uncovering key mechanisms underlying neuroproliferation and differentiation in Alzheimer's disease models.

**Conclusion:**

These findings highlight the potential of lipid‐based interventions in restoring neurogenesis and neuronal maturation in neurodegenerative conditions.

